# Cardiometabolic diseases, total mortality, and benefits of adherence to a healthy lifestyle: a 13-year prospective UK Biobank study

**DOI:** 10.1186/s12967-022-03439-y

**Published:** 2022-05-19

**Authors:** Chenjie Xu, Zhi Cao

**Affiliations:** 1 School of Public Health, Hangzhou Normal University, Hangzhou, China; 2 School of Public Health, Zhejiang University School of Medicine, Yuhangtang Road 866, Hangzhou, 310058 China

**Keywords:** Cardiometabolic disease, Diabetes, Cardiovascular disease, Healthy lifestyle, Mortality

## Abstract

**Background:**

Cardiometabolic disease (CMD) increases the risk of mortality, but the extent to which this can be offset by adherence to a healthy lifestyle is unknown. We aimed to investigate whether and to what extent a combination of healthy lifestyle is associated with lower risk of total mortality that related to CMD.

**Methods:**

Data for this prospective analysis was sourced from the UK Biobank with 356,967 participants aged 37 to 73 years between 2006 and 2010. Adherence to a healthy lifestyle was determined on the basis of four factors: no smoking, healthy diet, body mass index < 30 kg/m^2^, and regular physical activity. CMD was defined as any of incidence of diabetes, coronary heart disease and stroke at baseline. Cox proportional hazards models were used to calculate hazard ratios (HRs) and confidence intervals (CIs) of the associations of CMDs and lifestyle factors with total mortality.

**Results:**

During a median follow-up of 13 years, a total of 21,473 death events occurred. The multivariable-adjusted HRs of mortality were 1.49 (95% CI 1.53–1.56) for one, 2.17 (95% CI 2.01–2.34) for two, and 3.75 (95% CI 3.04–4.61) for three CMDs. In joint exposure analysis, compared with CMDs-free and a favorable lifestyle, the HRs of mortality were 2.57 (95% CI 2.38–2.78) for patients with CMDs plus an unfavorable lifestyle and 1.58 (95% CI 1.50–1.66) for those with CMDs plus a favorable lifestyle. A favorable lifestyle attenuates the CMDs-related risk of mortality by approximately 63%. The mortality risk of CMDs-free people but have unfavorable lifestyle was higher than those who have over one CMDs but have favorable lifestyle.

**Conclusion:**

The potential effect of an increasing number of CMDs on total mortality appears additive, adherence to a healthy lifestyle may attenuate the CMDs-related mortality risk by more than 60%. These findings highlight the potential importance of lifestyle interventions to reduce risk of mortality across entire populations, even in patients with CMDs.

**Supplementary Information:**

The online version contains supplementary material available at 10.1186/s12967-022-03439-y.

## Introduction

The prevalence of cardiometabolic diseases (CMDs), which defined as diabetes, coronary heart disease (CHD) and stroke, is increasing rapidly [1–3]. An analysis of data from the UK indicated that the combination of diabetes and cardiovascular diseases is the most common multimorbidity pattern [4]. The causes of death due to diabetes, CHD and stroke increased by 34.7%, 22.3% and 16.6% between 2007 and 2017 [5]. Considerable evidence has suggested that having any one of these conditions alone could increase the risk of mortality [6–9]. However, research regarding the magnitude of risk of mortality in patients with cardiometabolic multimorbidity concomitantly is sparse.

Lifestyle is an essential modifiable risk factor for risk of mortality. Emerging prospective studies have ascertained that adherence to a healthy lifestyle, including no smoking, regular physical activity, and healthy diet, was substantially associated with lower risk of mortality [10–12]. At the same time, these lifestyle risk factors often commonly coexist. However, previous studies did not identify whether and to what extent a combination of lifestyle factors were associated with the risk of mortality in patients with CMDs. Given the high prevalence of CMDs, it is of great public health and clinical prognosis significance to reduce the risk of mortality through intervention of combined healthy lifestyle.

It might be hypothesized that CMD significantly increases the risk of total mortality, but adherence to a healthy lifestyle can help compensate this risk. The current study aimed to determine whether there is evidence of a potential additive effect of three CMDs on total mortality, and further to explore whether and to what extent a healthy lifestyle may counteract the risk of mortality associated with CMD using the 11-year follow-up data from the UK Biobank cohort.

## Methods

### Study design and population

This is a prospective, population-based cohort study of participants enrolled in UK Biobank. Between April 2006 and December 2010, UK Biobank recruited 502,528 adults aged 40–69 years from the general population, participants attended one of 22 assessment centers across England, Scotland, and Wales, where they completed touchscreen and nurse-led questionnaires, had physical measurements taken, and provided biological samples [13]. Since recruitment, participants have been followed for mortality via death register record. All participants provided written informed consent and the study was approved by the NHS National Research Ethics Service (Ref: 11/NW/0382). Participants with prevalent cancer diagnosis identified at baseline via cancer register records were excluded. A total of 356,967 individuals who met the criteria were included in the final analysis (Additional file 1: Figure S1).

### Lifestyle factors

We defined four healthy lifestyle factors on the basis of the American Heart Association guidelines: no current smoking, a healthy diet, body mass index < 30 kg/m^2^, and moderate physical activity two or more times weekly [14, 15]. The UK Biobank participants completed a touch-screen questionnaire led by professionals on their usual diet pattern. In this analysis, a healthy diet was defined as adequate intake of at least 4 of 7 dietary components (fruits, vegetables, whole grains, refined grains, fish, unprocessed meat, and processed meat) in UK Biobank following the recommendations on dietary components for cardiovascular health [16–18]. Details of the assessments of diet pattern can be found in the Additional file 1: Text S1. Moderate physical activity was defined as at least 150 min of moderate intensity activity weekly or 75 min of vigorous activity weekly. Participants scored 1 point for each of 4 healthy lifestyle factors. The lifestyle index scores ranged from 0 to 4, with higher scores indicating higher adherence to healthy lifestyle, and were subsequently categorized as favorable (3 or 4 healthy lifestyle factors), intermediate (2 healthy lifestyle factors), and unfavorable (0 or 1 healthy lifestyle factor) lifestyles.

### Ascertainment of CMDs

CMDs included any of diabetes, CHD and stroke. Information on a baseline history of diabetes, stroke, and CHD was available by self-reported and hospital inpatient records (ICD-10 code: diabetes [E10, E11, E12, E13, E14], CHD [I20, I21, I22, I23, I24.1, I25.1, I25.2, I25.5, I25.6, I25.8, I25.9], stroke [I60, I61, I62, I63, I64]). UK Biobank undertook comprehensive data linkage for mortality status. Date and cause of death were obtained from the National Health Service (NHS) Information Centre for participants from England and Wales, and from the NHS Central Register, Scotland, for participants from Scotland. The date of the latest mortality follow-up was September 30, 2021.

### Covariates

Covariates including socio-demographics, behavioral and lifestyle factors, biological indicators, and comorbidities were adjusted in our analyses. Information on participants’ age, sex, ethnicity, qualifications, employment status, smoking status, alcohol intake frequency, hypertension, aspirin use, insulin use were collected using a self-reported questionnaire at baseline. The Townsend deprivation index was assigned as a continuous measure based on postcodes, which were derived from census data on housing, employment, social class and car availability, a higher index indicated more deprivation [19]. Blood samples were collected at baseline (2006–2010). Biomarkers, such as cholesterol, C-reactive protein and triglycerides, were measured by immunoturbidimetric assay on a Beckman Coulter AU5800. Details of the assessments of covariates can be found in the Additional file 1: Text S2.

### Statistical analyses

The characteristics of the sample are presented as mean ± standard deviation (SD) or percentage when appropriate across CMD numbers. Person-year was calculated from the date of recruitment to date of the death or censoring date, whichever occurred first.

Cox proportional hazard regression models with age as underlying timescale were utilized to estimate hazard ratios (HRs) and 95% CIs for the associations of CMDs status and lifestyle factors with total mortality. The proportional hazard assumption was checked by tests based on Schoenfeld residuals, and the results indicated that the assumptions had not been violated. The basic model was adjusted for age (timescale), sex, Townsend deprivation index, qualifications, employment, ethnicity, cholesterol, C-reactive protein, triglycerides, hypertension, use of aspirin, and use of insulin, and we further fitted an additional model that CMDs and lifestyle were adjusted for each other. To further investigate whether and to what extent healthy lifestyle can counteract the harmful effect of CMDs on mortality risk, we created a variable with 6 categories, which combines CMDs (no, yes) with the lifestyle factors (unfavorable, intermediate, favorable). Last but not least, to distinguish the beneficial effect of a favorable lifestyle on mortality reduction among people with and without CMDs, the associations between lifestyle factors and mortality stratified by CMDs were explored, and interaction effect between lifestyle factors and CMDs status was tested by included an interaction term in Cox model.

Subgroup analyses were further conducted to examine possible moderating effects of sex and age on the associations of CMDs status and lifestyle factors with mortality. Several sensitivity analyses were conducted to test the robustness of our results. We repeated all analyses after excluding all participants who died during the first 2 years of follow up to reduce the possibility of spurious associations due to reverse causation. We used a multiple imputation approach to impute the missing values for the non-systematically missing covariables. Five imputed datasets were generated and estimates were combined using Rubin’s rules. We further constructed a weighted lifestyle score on the four lifestyle factors by using the equation and repeated the main analyses: weighted healthy lifestyle score = (β_1_ × factor_1_ + β_2_ × factor_2_ + β_3_ × factor_3_ + β_4_ × factor_4_) × (4/sum of the β coefficients). This weighted lifestyle score has considered magnitudes of the adjusted relative risk for each lifestyle factor as a combination of four factors. In consideration of environmental factors have been implicated in CMDs, air pollution (such as PM 10, PM 2.5, and NO 2) was additionally adjusted in the multi-variable models. Meanwhile, family history of CHD, stroke and diabetes was further adjusted in the sensitivity analyses.

All analyses were performed using STATA 15 statistical software (StataCorp). All *P* values were two‐sided, and *P* < 0.05 was considered statistically significant.

## Results

Completed data in our final analysis were available for 356,967 participants in the UK Biobank. Table 1 shows the baseline characteristics of the study population. During a total of 356,967 person years (median follow-up 13.0 years), 21,473 death cases were occurred. At baseline, 29,859 (8.4%) participants had at least one CMD. Those with one, two, or three CMDs were more likely to be older and male than those with no CMDs.

**Table 1:** Baseline characteristics of the study population by the number of CMDs

Characteristics	Total (N = 356,967)	Number of CMDs			
		No (323,339)	One (29,859)	Two (3,571)	Three (198)
Age (mean, SD)	56.1 (8.1)	55.6 (8.1)	60.3 (6.9)	62.1 (5.8)	62.4 (6.0)
Female	187,615 (52.6)	176,147 (54.5)	10,545 (35.3)	890 (25.0)	46 (23.2)
Townsend deprivation index (mean, SD)	− 1.4 (3.0)	− 1.5 (3.0)	− 0.9 (3.3)	− 0.325 (3.5)	0.4 (3.6)
Ethnicity					
White	337,288 (94.5)	306,630 (94.8)	27,295 (91.4)	3,195 (89.5)	168 (84.8)
Mixed background	2,147 (0.6)	1,984 (0.6)	145 (0.5)	13 (0.4)	5 (2.5)
Asian or Asian British	6,554 (1.8)	5,154 (1.6)	1,165 (3.9)	223 (6.2)	12 (6.1)
Black or black British	5694 (1.6)	4,902 (1.5)	713 (2.4)	77 (2.2)	2 (1.0)
Chinese	1116 (0.3)	1,035 (0.3)	77 (0.3)	4 (0.1)	0 (0.0)
Others	3101 (0.9)	2,693 (0.8)	354 (1.2)	47 (1.3)	7 (3.5)
Missing	1067 (0.3)	941 (0.3)	110 (0.4)	12 (0.3)	4 (2.0)
Employment					
Working	213,316 (59.8)	200,608 (62.0)	11,756 (39.4)	922 (25.8)	30 (15.1)
Retired	114,442 (32.1)	97,421 (30.1)	14,884 (49.8)	2,021 (56.6)	116 (58.6)
Unemployed	23,557 (6.6)	20,138 (6.2)	2,793 (9.3)	580 (16.2)	46 (23.2)
Other	5,652 (1.6)	5,172 (1.6)	426 (1.4)	48 (1.3)	6 (3.0)
Qualifications					
College degree	125,987 (35.3)	117,544 (36.3)	7,691 (25.8)	706 (19.8)	46 (23.2)
A levels/AS levels	41,269 (11.6)	38,078 (11.8)	2,879 (9.6)	299 (8.4)	14 (7.1)
O levels/GCESs	74,858 (21.0)	68,355 (21.1)	5,794 (19.4)	677 (19.0)	32 (16.2)
CSEs	19,135 (5.4)	17,688 (5.5)	1,300 (4.3)	142 (4.0)	5 (2.5)
NVQ or HND or HNC	23,648 (6.6)	20,602 (6.4)	2,640 (8.8)	393 (11.0)	13 (6.6)
Other	18,314 (5.1)	16,260 (5.0)	1,835 (6.1)	209 (5.89)	10 (5.0)
None of the above	51,036 (14.3)	42,518 (13.1)	7,359 (24.6)	1,085 (30.4)	74 (37.4)
Missing	2.720 (0.8)	2294 (0.7)	362 (1.2)	60 (1.7)	4 (2.0)
Cholesterol (mmol/L; mean, SD)	5.7 (1.1)	5.8 (1.0)	4.7 (1.1)	4.4 (1.0)	4.4 (1.1)
C-reactive protein (mg/L; mean, SD)	2.4 (3.9)	2.3 (3.8)	2.8 (4.6)	3.3 (5.2)	3.4 (4.6)
Triglycerides (mmol/L; mean, SD)	1.7 (1.0)	1.7 (1.0)	1.9 (1.1)	2.1 (1.2)	2.2 (1.4)
Lifestyle factors					
Healthy diet	224,192 (62.8)	203,894 (63.1)	18,127 (60.7)	2075 (58.1)	96 (48.5)
Regular physical activity	220,888 (61.9)	200,856 (62.1)	17,873 (59.9)	2049 (57.4)	110 (55.6)
Non-smoker	322,200 (90.3)	292,178 (90.4)	26,720 (89.5)	3134 (87.8)	168 (84.8)
BMI < 30 kg/m^2^	277,672 (77.8)	257,756 (79.7)	18,099 (60.6)	1,736 (48.6)	81 (40.9)

Values are numbers (percentages) unless stated otherwise

BMI, body mass index (calculated as weight in kilograms divided by height in meters squared); CSE, Certificate of Secondary Education; GCSE, General Certificate of Secondary Education; HNC, Higher National Certificate; HND, Higher National Diploma; NVQ, National Vocational Qualification; SD, standard deviation

The mortality rates per 1000 person-year were 4.0 (95% CI 3.9–4.1) in participants without CMDs, 23.0 (95% CI 21.6–24.5) in those with more than one CMDs (Table 2). When we adjusted for potential confounders, the HRs of mortality in patients who had one or more CMDs were 1.56 (95% CI 1.50–1.63). The risk of mortality increased monotonically with increased CMDs number. The HRs of mortality were 1.50 (95% CI 1.44–1.56) for one, 2.19 (95% CI 2.03–2.36) for two, and 3.90 (95% CI 3.17–4.80) for three CMDs. After adjustment for lifestyle factors (model 2), the associations between CMDs status and mortality were slightly attenuated. The number of CMDs was dose-dependently associated with increased risk for mortality (*P* for trend < 0.001).

**Table 2:** Mortality rate and hazard ratios with 95% CIs of total mortality in relation to CMDs status

CMDs status	Events	Person-years	Mortality rate per 1000 person-year	Hazard ratio (95% CI)	
				Model 1^b^	Model 2 ^c^
No	16,287	4,055,268	4.0 (3.9–4.1)	1 (Ref.)	1 (Ref.)
Yes	5186	404,587	12.8 (12.5–13.2)	1.57 (1.51–1.64)	1.56 (1.50–1.63)
Only one	4152	361,739	11.5 (11.1–11.8)	1.50 (1.44–1.56)	1.49 (1.43–1.56)
Diabetes	1655	154,457	10.7 (10.2–11.2)	1.50 (1.41–1.59)	1.48 (1.40–1.57)
Stroke	561	51,280	10.9 (10.1–11.9)	1.56 (1.43–1.70)	1.52 (1.40–1.66)
CHD	1936	156,002	12.4 (11.9–13.0)	1.48 (1.41–1.57)	1.49 (1.41–1.58)
Any two	940	40,809	23.0 (21.6–24.5)	2.19 (2.03–2.36)	2.17 (2.01–2.34)
CHD & stroke	175	8088	21.6 (18.0–26.1)	2.13 (1.83–2.48)	2.11 (1.81–2.45)
Diabetes & stroke	112	5167	21.7 (18.0–26.1)	2.17 (1.80–2.62)	2.15 (1.78–2.60)
Diabetes & CHD	653	27,553	23.7 (21.9–25.6)	2.20 (2.01–2.41)	2.20 (2.01–2.40)
Three (Diabetes & CHD & stroke)	94	2040	46.1 (37.6–56.4)	3.90 (3.17–4.80)	3.75 (3.04–4.61)
*P* for trend ^a^				< 0.001	< 0.001

CHD, coronary heart disease; CMD, cardiometabolic disease

^a^*P* value of linear trend was calculated by included the number of CMDs as a continuous variable in the model

^b^Model 1 adjusted for age, sex, ethnicity, Townsend deprivation index, qualifications, employment, cholesterol, C-reactive protein, triglycerides, hypertension, use of aspirin, and use of insulin

^c^Model 2 further adjusted for lifestyle

**Fig. 1:**
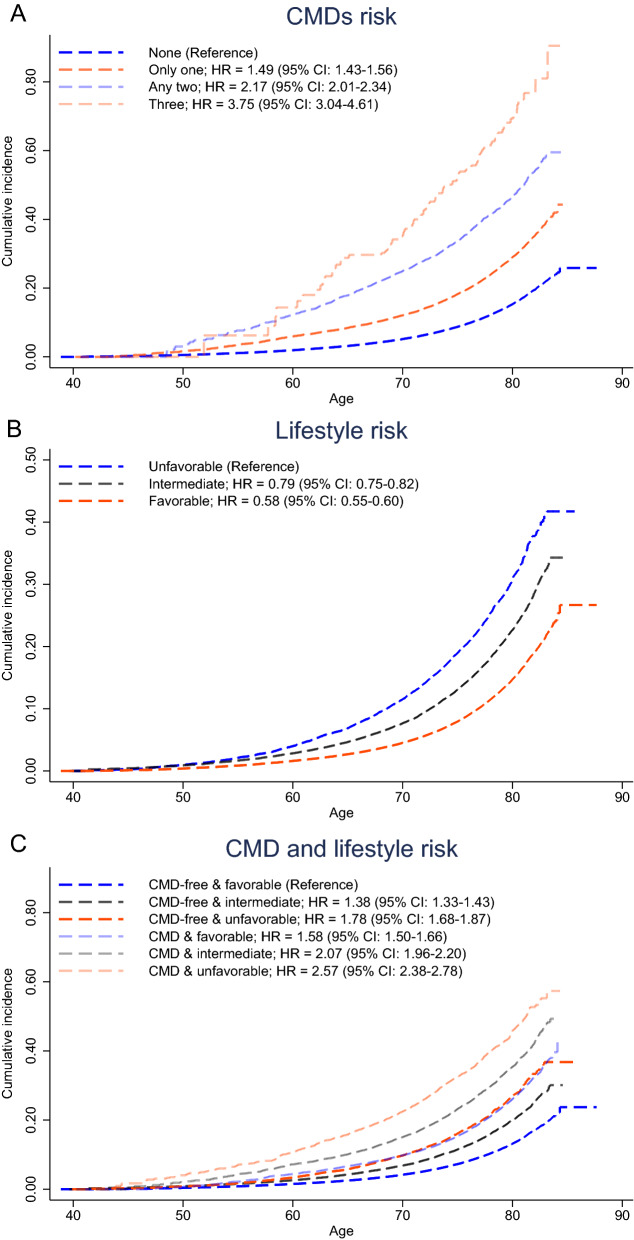
Cumulative incidence and hazard ratios of total mortality according to CMDs status and lifestyle categories. The association of CMDs status (**A**), lifestyle (**B**), combined CMDs and lifestyle (**C**) with total mortality. Cox regression models were adjusted for age, sex, ethnicity, Townsend deprivation index, qualifications, employment, cholesterol, C-reactive protein, triglycerides, hypertension, use of aspirin, and use of insulin

The risk of mortality was reduced in those with a favorable lifestyle (HR 0.58, 95% CI 0.55–0.60) and intermediate lifestyle (HR 0.79, 95% CI 0.75–0.82) compared with those with an unfavorable lifestyle. The cumulative mortality rate of unfavorable lifestyle category was obviously higher than intermediate and favorable lifestyle (Fig. 1). The same pattern of results was observed when the number of healthy lifestyle factors was used instead of lifestyle categories (Additional file 1: Table S1). In addition, each of the four healthy lifestyles (healthy diet, regular physical activity, no smoking, BMI < 30 kg/m^2^) was associated with 7%-53% lower risk of mortality (Additional file 1: Table S2).

**Fig. 2:**
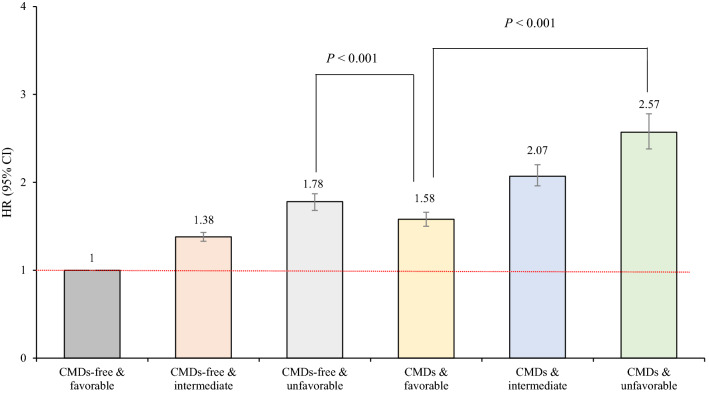
Joint effect of CMDs status and lifestyle factors on risk of total mortality. Cox regression models were adjusted for age, sex, ethnicity, Townsend deprivation index, qualifications, employment, cholesterol, C-reactive protein, triglycerides, hypertension, use of aspirin, and use of insulin

When CMDs status and lifestyle factors categories were combined, there was a monotonic association with increasing CMDs risk and an increasingly unhealthy lifestyle (Fig. 2). The multivariable-adjusted HRs of mortality were 2.57 (95% CI 2.38–2.78) for patients with CMDs and an unfavorable lifestyle and 1.58 (95% CI 1.50– 1.66) for those with CMDs and a favorable lifestyle, compared with CMDs-free and favorable lifestyle. Moreover, we found that the mortality risk of CMDs-free people but have unfavorable lifestyle was significantly higher than with those who have over one CMD but have favorable lifestyle (*P* < 0.001) (Additional file 1: Table S3).

Further analyses stratified by CMDs status category with unfavorable lifestyle as the reference group confirmed that a favorable lifestyle was associated with a lower mortality risk regardless of CMDs status (Table 3). Among patients with CMDs, the mortality rates per 1000 person-year were 17.7 (95% CI 16.5–18.9) among those with an unfavorable lifestyle and 11.1 (95% CI 10.7–11.5) among those with a favorable lifestyle, and adherence to a favorable lifestyle, as compared with an unfavorable lifestyle, was associated with a lower relative risk among those at intermediate lifestyle (HR = 0.81, 95% CI 0.75–0.89) and favorable lifestyle (HR = 0.62, 95% CI 0.57–0.67). There was no significant interaction between CMDs and lifestyle factors in relation to mortality risk (*P* for interaction = 0.125).

**Table 3:** Risk of total mortality according to lifestyle factors category within CMDs status

CMDs status	Lifestyle	Events	Person-years	Mortality rate^a^	HR (95% CI)^b^
CMD	Unfavorable	823	46,553	17.7 (16.5–18.9)	1 (Ref.)
	Intermediate	1685	116,571	14.4 (13.9–15.2)	0.81 (0.75–0.89)
	Favorable	2678	241,464	11.1 (10.7–11.5)	0.62 (0.57–0.67)
CMD-free	Unfavorable	1684	264,125	6.4 (6.1–6.7)	1 (Ref.)
	Intermediate	4395	911,289	4.8 (4.7–5.0)	0.77 (0.73–0.82)
	Favorable	10,208	2,879,854	3.5 (3.5–3.6)	0.56 (0.53–0.59)

^a^Mortality rate per 1000 person-years

^b^Cox regression models were adjusted for age, sex, ethnicity, Townsend deprivation index, qualifications, employment, cholesterol, C-reactive protein, triglycerides, hypertension, use of aspirin, and use of insulin

We further explored the stratified and combined associations of CMDs status and lifestyle factors with total mortality by sex and age subgroups. Results have not changed substantially. The association of CMDs and lifestyle with mortality was not varied across sex (*P* for interaction > 0.05) **(**Additional file 1: Table S4**)**, and there were significant interactions between CMDs or lifestyle and age in relation to mortality **(**Additional file 1: Table S5**)**. In addition, the joint risk of CMDs and unfavorable lifestyle differed by different age subgroups (*P* for interaction < 0.001) (Additional file 1: Table S6 and Table S7). In the sensitivity analyses, we repeated the main analysis among participants with at least 2 years of follow-up, and similar results were observed (Additional file 1: Table S8**)**. Results of associations of CMDs status and lifestyle factors with total mortality with multiple imputed data showed that the HRs remained significant (Additional file 1: Table S9). The results were not materially changed for weighted healthy lifestyle score (Additional file 1: Table S10). Additional file 1: Table S11 and S12 shows the HRs of mortality by adjusting for air pollution and family history of CMDs, respectively.

## Discussion

In this large-scale population-based cohort study, we found that CMDs were associated with higher risk of total mortality, and the risk was dose-dependently related to the number of CMDs, there is an additive effect of three CMDs on total mortality. Furthermore, adherence to a healthy lifestyle was associated with a lower risk of mortality regardless of CMDs status, and the detrimental effect of CMDs on mortality might be offset by adherence to a healthy lifestyle by 63%. In addition, we found that the mortality of CMDs-free people but have unfavorable lifestyle was higher than those who have over one CMDs but have a favorable lifestyle.

In our study, in patients who had only 1 CMD condition, we observed a HR for mortality of 1.49; for a combination of any 2 conditions, the HR was 2.17; and for a combination of all 3 conditions, the HR was 3.75. These results revealed that there was an additive effect of the association between CMDs and mortality. Given the clustering of different CMDs, it is important to elucidate the combined contribution of CMDs to risk of mortality. A large-scale study of 474,129 participants from UK Biobank found that CMDs might contribute to cognitive decline, and the potential effect of an increasing number of CMDs consistently appeared additive [20]. Consequently, our findings emphasized the great significance of prevention for cardiovascular disease in patients who already have prevalent diabetes, conversely, to avert diabetes in patients who already have prevalent cardiovascular disease.

To the best of our knowledge, prior study has not examined the association of a combination of lifestyle factors and CMDs status with risk of mortality. Our findings showed that prevalent CMDs combined with an unfavorable lifestyle profile was associated with a more than twofold higher risk of mortality compared with CMDs-free and a favorable lifestyle. A previous study from Nurses’ Health Study and Health Professionals Follow-up Study in US showed that greater improvements in healthy lifestyle factors were significantly associated with a lower risk of CVD incidence or CVD mortality [12]. A systematic review and meta-analysis suggested that adherence to a healthy lifestyle (including no smoking, moderate alcohol intake, leisure-time physical activity, healthy weight) may separately reduce the risk of mortality among middle-aged and elderly women [21]. Even though for patients after being diagnosed with stroke, combinations of five healthy lifestyle factors (fruits and vegetables intake, no smoking, moderate alcohol intake, exercising, healthy weight) were associated with lower all-cause and cardiovascular disease mortality in a dose dependent fashion [22]. However, differed from the patients with CVD or diabetes, our study highlighted the risk of mortality among general population, and we found that the harmful effect of mortality associated with the combination of CHD, stroke and diabetes might be partially offset by a healthy lifestyle.

Diet is one of the most important modifiable risk factors that could represent a good and relatively easy point of intervention to reduce the risk of CVD [23]. A healthy diet pattern potentially interactive and cumulative associations of different dietary components, and could reflect the numerous and multifaceted combinations of nutrient and food consumption [24]. Several published observational studies have found a beneficial effect of greater adherence to a healthy diet pattern on CMDs [25–28]. Randomised controlled trials have also strengthened the beneficial impact of physical exercise and healthy diet on cardiorespiratory fitness, and cardiometabolic health in adults [29, 30]. A fundamental feature of cardiac metabolism is its flexibility. Lifestyle changes are very effective in improving the quality of life among individuals with existing CMDs [23].

The protective effect of healthy lifestyle on CMDs can be explained by genetic pathways. One previous study using a polygenic risk score suggested that a favorable lifestyle was associated with a nearly 50% lower relative risk of coronary artery disease among participants at high genetic risk [31]. Another epidemiological study also found that adherence to a healthy lifestyle might attenuate the risk of stroke independently of genetic risk [15]. A poor lifestyle was associated with 15-fold higher risk of diabetes in high genetic risk, ideal lifestyle profile could attenuate the risk of incident diabetes among any genetic risk groups [17]. These findings indicated the strong potential great benefits of adherence to healthy behavioral lifestyle factors regardless of genetic risk. Not only does a healthy lifestyle reduce the risk of developing CMDs, it also directly reduces the risk of mortality. Healthy lifestyle interventions are important to reduce risk of mortality across entire populations, even in patients with CMDs. In addition, the associations between CMDs and mortality was modified by age. The association of combined CMDs and lifestyle with risk of mortality in population aged < 60 years was stronger than that in older individuals. It is consistent with the findings that improvement of a composite metric of cardiovascular health (including exercise, cigarette smoking, BMI, etc.) could reduce risk of cardiovascular disease for the middle and aged populations [32].

Notable strengths of the present study included its prospective design and relatively large sample size, which provided us with adequate statistical power to evaluate the associations of CMDs status and lifestyle factors with total mortality. Despite these strengths, several limitations of our study need to be considered. First, CMDs only included cardiovascular diseases [33, 34] but no other vascular risk factors, such as high cholesterol and hypertension, because categorizing these risk factors as a binary variable would underestimate the true effect of high cholesterol and hypertension on chronic disease, rather, high cholesterol and blood pressure were adjusted as confounders in our study. Second, although analyses were adjusted for a wide of potential confounders and participants were followed up for a median of 13 years, the possibility of unmeasured con Additional file: As per journal requirements, every additional file must have a corresponding caption. In this regard, please be informed that the caption was taken from the additional e-file itself. Please advise if action taken appropriate and amend if necessary.founding and reverse causation remains. Third, we limited our analyses to healthy diet that only included 7 common food types. Information on total caloric load, intake of plant polyphenols, omega 3 fatty acids was not taken into account. Finally, we did not have access to time-varying exposure information to enable updating multimorbidity status and covariates during follow-up.

## Conclusion

In the current population-based cohort study, we found that prevalent CMDs were associated with higher risk of mortality, and the risk increased in an additive dose-dependent fashion. However, adherence to a healthy lifestyle may significantly offset the risk of mortality related to CMDs. Our findings emphasized the public health priority for healthy lifestyle of both primary prevention of individual CMDs and secondary prevention among patients with one CMD who are at risk of developing a comorbid disease.

## Supplementary Information

**Additional file 1: Text S1 **Diet score extended information. **Text S2 **Variable assessment. **Table S1.** Risk of all-cause mortality according to number of lifestyle factors. **Table S2.** The association of separate lifestyle factors with total mortality. **Table S3.** Mortality rate and hazard ratio of CMDs status and lifestyle factors in relation to mortality. **Table S4.** The association of CMDs status and lifestyle factors with total mortality in subgroup of sex. **Table S5.** The association of CMDs status and lifestyle factors with total mortality in subgroup of age. **Table S6.** Joint effect of CMDs and lifestyle factors on total mortality in subgroup of sex. **Table S7.** Joint effect of CMDs status and lifestyle factors on total mortality in subgroup of age. **Table S8.** Hazard ratios for cancers after exclusion of first two years of follow-up across CMDs status and lifestyle factors. **Table S9**. Joint associations of CMDs status and lifestyle factors with total mortality when missing covariates are imputed by multiple imputation. **Table S10**. Risk of all-cause mortality according to lifestyle factors category within CMDs status by constructing a weighted lifestyle score. **Table S11**. Risk of all-cause mortality according to lifestyle factors category within CMDs status after adjusting PM 10, PM 2.5, and NO_2_. **Table S12.** Risk of all-cause mortality according to lifestyle factors category within CMDs status after adjusting family history of CMDs. **Figure S1.** Participant flow diagram.

## Data Availability

The data that support the findings of this study are available from UK Biobank project site, subject to registration and application process. Further details can be found at https://www.ukbiobank.ac.uk.
