# Supplementary material for: Cardiometabolic diseases, total mortality, and benefits of adherence to a healthy lifestyle: a 13-year prospective UK Biobank study
Source: J Transl Med. 2022 May 19;20:234. doi: 10.1186/s12967-022-03439-y (PMC9118619; doi:10.1186/s12967-022-03439-y)
Supplement: Supplementary file 1 — Additional file 1: Text S1 Diet score extended information. Text S2 Variable assessment. Table S1. Risk of all-cause mortality according to number of lifestyle factors. Table S2. The association of separate lifestyle factors with total mortality. Table S3. Mortality rate and hazard ratio of CMDs status and lifestyle factors in relation to mortality. Table S4. The association of CMDs status and lifestyle factors with total mortality in subgroup of sex. Table S5. The association of CMDs status and lifestyle factors with total mortality in subgroup of age. Table S6. Joint effect of CMDs and lifestyle factors on total mortality in subgroup of sex. Table S7. Joint effect of CMDs status and lifestyle factors on total mortality in subgroup of age. Table S8. Hazard ratios for cancers after exclusion of first two years of follow-up across CMDs status and lifestyle factors. Table S9. Joint associations of CMDs status and lifestyle factors with total mortality when missing covariates are imputed by multiple imputation. Table S10. Risk of all-cause mortality according to lifestyle factors category within CMDs status by constructing a weighted lifestyle score. Table S11. Risk of all-cause mortality according to lifestyle factors category within CMDs status after adjusting PM 10, PM 2.5, and NO2. Table S12. Risk of all-cause mortality according to lifestyle factors category within CMDs status after adjusting family history of CMDs. Figure S1. Participant flow diagram. [file 12967_2022_3439_MOESM1_ESM.doc]

**Supplementary material**

**Text S1 Diet score extended information**

During the baseline assessment all UK Biobank participants completed an extensive questionnaire that included dietary habits (“Touchscreen Questionnaire”, available from http://www.ukbiobank.ac.uk/resources/).

UK Biobank Food Frequency Questionnaire at baseline; Healthy diet was defined based on at least 4 of the following 7 food groups:
1. Fruits: ≥ 3 servings/day
2. Vegetables: ≥ 3 servings/day
3. Fish: ≥2 servings/week
4. Processed meats: ≤ 1 serving/week
5. Unprocessed red meats: ≤ 2 servings/week
6. Whole grains: ≥3 servings/day
7. Refined grains: ≤ 2 servings/day

**Text S2:**

**Variable assessment**

Sociodemographic characteristics

Participants provided detailed self-reported data via a touch screen questionnaire at the assessment centres at baseline. Socio-demographic factors included age at recruitment (month and year of birth as acquired by the central registry and updated by participant, with each participant nominally assigned 15 as the day of birth), ethnicity (participants were asked “What is your ethnic group?” with possible answers being “White”, “Mixed”, “Asian or Asian British”, “Black or “Black British”, “Chinese”, “Other ethnic group”, “Do not know”, “Prefer not to answer”), qualifications (“Which of the following qualifications do you have?” with possible answers being “College degree”, “A levels/AS levels”, “O levels/GCESs”, “CSEs”, “NVQ or HND or HNC”, “Other professional qualifications”, “Do not know”, “Prefer not to answer”), employment (possible answers classify as “Working”, “retired”, “unemployment”, “other”), Townsend deprivation index was assigned based on postcode as a continuous measure, which derived from census data on housing, employment, social class and car availability, a higher index indicates more deprivation.

Anthropometric measurements

Anthropometric measurements were taken by trained research clinic staff (UK-Biobank, 2014). Standing height was measured to the nearest centimetre (cm), without shoes, using the SECA 240 Height Measure. Weight measurement was measured without shoes and outdoor clothing, using the Tanita BC 418 body composition analyser or using standard scales if the participant did not undergo bioimpedance analysis. Body mass index (BMI) was calculated as weight (kg)/height (m^2^). Percentage body fat was measured using the Tanita BC 418 body composition analyser. Waist circumference were collected from participants in a horizontal plane using a Seca 200cm tape measure. Systolic/diastolic blood pressure were calculated from the second measurement of the two baseline recordings.

Blood Analysis

Blood was collected from all participants by venepuncture into EDTA coated tubes during the UK Biobank assessment centre visit. A Beckman Coulter LH750 haematology analyser and leukocyte counter was used to measure total and differential blood cell counts within 24h of sample collection. The neutrophil to lymphocyte ratio was calculated from the total numbers of neutrophils and lymphocytes per litre, and taken as an index of inflammation (immune activation).

Lifestyle characteristics

Smoking status was self-reported, and based on current/past smoking status the participant was classified as never, former, or current smoker. Total physical activity was computed as the sum of walking (2.3 excess metabolic equivalents [METs]), moderate activity (3.0 excess METs) and vigorous activity (7.0 excess METs) (for at least 10 minutes continuously). We report excess METs, which represent the energy expenditure above that of an inactive person.

Alcohol intake was asked participants how often they drank alcohol with the possible answers being: “daily or almost daily”, “three or four times a week”, “once or twice a week”, “one to three times a month”, “special occasions only”, “never”, “prefer not to answer”. If the participant reported ‘do not know’ or ‘prefer not to answer’ to one of these questions on weekly or monthly consumption, they were coded as missing.

For fruit, participants were asked to direct enter the number of pieces of fresh fruit and dried fruit (with examples given as to what constitutes a piece eaten per day) or select ‘less than one’, ‘do not know’ or ‘prefer not to answer’. One piece of fresh fruit, and two ‘pieces’ of dried fruit were counted as a serving. We grouped participants into the following categories: <2.0 servings per day, 2.0-2.9 servings per day, 3.0-3.9 servings per day, and ≥4.0 servings per day.

For vegetables, participants were asked to direct enter the number of heaped tablespoons of cooked vegetables and salad/raw vegetables eaten per day or select ‘less than one’, ‘do not know’ or ‘prefer not to answer’. Two heaped tablespoons of vegetables were counted as a serving. We grouped participants into the following categories: <2.0 servings per day, 2.0-2.9 servings per day, 3.0-3.9 servings per day, and ≥4.0 servings per day.

For processed meat, poultry, oily fish and non-oily fish, we combined the top three frequencies to get four categories: never, <1.0 time per week, 1.0 time per week, and ≥2.0 times per week’. To rank the participants by weekly red meat consumption based on the touchscreen, we summed the frequencies for beef, pork, and lamb/mutton, using the following coding: ‘Never’ = 0, ‘Less than once a week’ = 0.5, ‘Once a week’ = 1, ‘2-4 times a week’ = 3, ‘5-6 times a week’ = 5.5, ‘Once or more daily’ = 7. The four categories for red meat consumption were: <1 time per week, 1.0-1.9 times per week, 2.0-2.9 times per week, and ≥3.0 times per week.

For red and processed meat, we summed the frequencies for beef, pork, lamb/mutton, and processed meat. The categories for red and processed meat consumption were: < 2.0 times per week, 2.0-2.9 times per week, 3.0-3.9 times per week, and ≥4.0 times per week. To rank the participants by weekly total fish consumption based on the touchscreen, we summed the frequencies for oily fish and non-oily fish and grouped participants into the following categories: <1.0 time per week, 1.0-1.9 times per week, 2.0-2.9 times per week, and ≥3.0 times per week.

Participants were asked to report their level and duration of usual physical activity ranked by intensity (vigorous, moderate and walking), and these data were used to derive total physical activity measured as metabolic equivalents (MET-hours/week), as described by the International Physical Activity Questionnaire (IPAQ) scoring protocol.

Further details of these measurements can be found in the UK Biobank online protocol (http://www.ukbiobank.ac.uk).

Table S1: Risk of all-cause mortality according to number of lifestyle factors.

| Healthy lifestyle scores | HR (95% CI) | P value |
| --- | --- | --- |
| 0 healthy lifestyle factor | 1 (Ref.) |  |
| 1 healthy lifestyle factor | 0.67 (0.59-0.76) | <0.001 |
| 2 healthy lifestyle factors | 0.54 (0.48-0.61) | <0.001 |
| 3 healthy lifestyle factors | 0.42 (0.37-0.48) | <0.001 |
| 4 healthy lifestyle factors | 0.36 (0.32-0.41) | <0.001 |
| P for trend | <0.001 | |

The analyses were adjusted for age, sex, ethnicity, Townsend deprivation index, qualifications, employment, cholesterol, C-reactive protein, triglycerides, hypertension, use of aspirin, use of insulin, and CMDs.

Table S2: The association of separate lifestyle factors with total mortality.

| Lifestyle | Events | Person-years | Mortality rate (95% CI) | Hazard ratio (95% CI) | |
| --- | --- | --- | --- | --- | --- |
|  |  |  |  | Model 1† | Model 2‡ |
| Healthy diet | 12,130 | 2,808,180 | 4.3 (4.2-4.4) | 0.86 (0.83-0.88) | 0.85 (0.83-0.88) |
| Regular physical activity | 13,164 | 2,758,522 | 4.8 (4.7-4.8) | 0.93 (0.90-0.96) | 0.93 (0.91-0.96) |
| Non-smoker | 17,602 | 426,397 | 4.4 (4.3-4.4) | 0.47 (0.46-0.49) | 0.47 (0.45-0.49) |
| BMI < 30 kg/m^2^ | 15,234 | 3475374 | 4.4 (4.3-4.5) | 0.84 (0.82-0.87) | 0.86 (0.84-0.89) |

† Model 1 adjusted for age, sex, ethnicity, Townsend deprivation index, qualifications, employment, cholesterol, C-reactive protein, triglycerides, hypertension, use of aspirin, and use of insulin.

‡ Model 2 further adjusted for CMDs.

Table S3: Mortality rate and hazard ratio of CMDs status and lifestyle factors in relation to mortality.

| CMDs status | Lifestyle | Events | Person-year | Mortality rate per 1000 person-years | HR (95% CI) |
| --- | --- | --- | --- | --- | --- |
| No | Favorable | 10208 | 2,879,854 | 3.5 (3.4-3.6) | 1 (Ref.) |
|  | Intermediate | 4,395 | 911,289 | 4.8 (4.7-5.0) | 1.38 (1.33-1.43) |
|  | Unfavorable | 1,684 | 264,125 | 6.4 (6.1-6.7) | 1.78 (1.68-1.87) |
| Yes | Favorable | 2,678 | 241,464 | 11.1 (10.7-11.5) | 1.58 (1.50-1.66) |
|  | Intermediate | 1,685 | 116,571 | 14.4 (13.8-15.2) | 2.07 (1.96-2.20) |
|  | Unfavorable | 823 | 46,553 | 17.7 (16.5-18.9) | 2.57 (2.38-2.78) |

Cox regression models were adjusted for age, sex, ethnicity, Townsend deprivation index, qualifications, employment, cholesterol, C-reactive protein, triglycerides, hypertension, use of aspirin, and use of insulin.

Table S4: The association of CMDs status and lifestyle factors with total mortality in subgroup of sex.

|  | Male | Female | P for interaction |
| --- | --- | --- | --- |
| CMDs |  |  | 0.378 |
| None | 1 (Ref.) | 1 (Ref.) |  |
| Only one | 1.49 (1.42-1.57) | 1.50 (1.39-1.62) |  |
| Any two | 2.17 (1.99-2.37) | 2.18 (1.85-2.58) |  |
| Three | 3.46 (2.73-4.39) | 4.71 (3.05-7.29) |  |
| Lifestyle |  |  | 0.135 |
| Unfavorable | 1 (Ref.) | 1 (Ref.) |  |
| Intermediate | 0.81 (0.77-0.86) | 0.73 (0.67-0.80) |  |
| Favorable | 0.59 (0.56-0.62) | 0.56 (0.519-0.60) |  |

The analyses were adjusted for age, sex, ethnicity, Townsend deprivation index, qualifications, employment, cholesterol, C-reactive protein, triglycerides, hypertension, use of aspirin, use of insulin, as well as CMDs and lifestyle were adjusted for each other.

Table S5: The association of CMDs status and lifestyle factors with total mortality in subgroup of age.

|  | Age <60 | Age ≥60 | P for interaction |
| --- | --- | --- | --- |
| CMDs |  |  | <0.001 |
| None | 1 (Ref.) | 1 (Ref.) |  |
| Only one | 1.60 (1.46-1.75) | 1.47 (1.40-1.54) |  |
| Any two | 2.30 (1.93-2.73) | 2.18 (2.00-2.37) |  |
| Three | 3.89 (2.35-6.44) | 3.88 (3.09-4.87) |  |
| Lifestyle |  |  | 0.031 |
| Favorable | 1 (Ref.) | 1 (Ref.) |  |
| Intermediate | 0.77 (0.71-0.83) | 0.81 (0.76-0.85) |  |
| Unfavorable | 0.57 (0.53-0.62) | 0.59 (0.56-0.62) |  |

The analyses were adjusted for age, sex, ethnicity, Townsend deprivation index, qualifications, employment, cholesterol, C-reactive protein, triglycerides, hypertension, use of aspirin, use of insulin, as well as CMDs and lifestyle were adjusted for each other.

Table S6: Joint effect of CMDs and lifestyle factors on total mortality in subgroup of sex.

| CMDs status | Lifestyle | Male | Female | P for interaction |
| --- | --- | --- | --- | --- |
| No | Favorable | 1 (Ref.) | 1 (Ref.) | 0.363 |
|  | Intermediate | 1.40 (1.34-1.47) | 1.33 (1.25-1.41) |  |
|  | Unfavorable | 1.76 (1.65-1.88) | 1.82 (1.66-1.99) |  |
| Yes | Favorable | 1.59 (1.50-1.69) | 1.58 (1.45-1.73) |  |
|  | Intermediate | 2.12 (1.98-2.27) | 1.94 (1.73-2.18) |  |
|  | Unfavorable | 2.54 (2.32-2.78) | 2.68 (2.28-3.15) |  |

The analyses were adjusted for age, sex, ethnicity, Townsend deprivation index, qualifications, employment, cholesterol, C-reactive protein, triglycerides, hypertension, use of aspirin, and use of insulin.

Table S7: Joint effect of CMDs status and lifestyle factors on total mortality in subgroup of age.

| CMDs status | Lifestyle | Age <60 | Age ≥60 | P for interaction |
| --- | --- | --- | --- | --- |
| No | Favorable | 1 (Ref.) | 1 (Ref.) | <0.001 |
|  | Intermediate | 1.36 (1.28-1.45) | 1.38 (1.32-1.44) |  |
|  | Unfavorable | 1.74 (1.59-1.89) | 1.78 (1.66-1.90) |  |
| Yes | Favorable | 1.67 (1.49-1.88) | 1.57 (1.48-1.66) |  |
|  | Intermediate | 2.09 (1.84-2.37) | 2.08 (1.95-2.23) |  |
|  | Unfavorable | 2.87 (2.50-3.31) | 2.42 (2.20-2.66) |  |

The analyses were adjusted for age, sex, ethnicity, Townsend deprivation index, qualifications, employment, cholesterol, C-reactive protein, triglycerides, hypertension, use of aspirin, and use of insulin.

**Table S8: Hazard ratios for cancers after exclusion of first two years of follow-up across CMDs status and lifestyle factors.**

| CMDs status | Lifestyle | HR (95% CI) |
| --- | --- | --- |
| No | Favorable | 1 (Ref.) |
|  | Intermediate | 1.38 (1.33-1.43) |
|  | Unfavorable | 1.80 (1.71-1.90) |
| Yes | Favorable | 1.56 (1.48-1.64) |
|  | Intermediate | 2.04 (1.92-2.16) |
|  | Unfavorable | 2.54 (2.35-2.76) |

The analyses were adjusted for age, sex, ethnicity, Townsend deprivation index, qualifications, employment, cholesterol, C-reactive protein, triglycerides, hypertension, use of aspirin, and use of insulin.

**Table S9: Joint associations of CMDs status and lifestyle factors with total mortality when missing covariates are imputed by multiple imputation.**

| CMDs status | Lifestyle | HR (95% CI) |
| --- | --- | --- |
| No | Favorable | 1 (Ref.) |
|  | Intermediate | 1.37 (1.32-1.42) |
|  | Unfavorable | 1.77 (1.68-1.87) |
| Yes | Favorable | 1.57 (1.49-1.65) |
|  | Intermediate | 2.05 (1.93-2.18) |
|  | Unfavorable | 2.54 (2.35-2.74) |

The analyses were adjusted for age, sex, ethnicity, Townsend deprivation index, qualifications, employment, cholesterol, C-reactive protein, triglycerides, hypertension, use of aspirin, and use of insulin.

Table S10: Risk of all-cause mortality according to lifestyle factors category within CMDs status by constructing a weighted lifestyle score.

| CMDs status | Lifestyle | HR (95% CI) ^b^ |
| --- | --- | --- |
| CMD | Unfavorable | 1 (Ref.) |
|  | Intermediate | 0.76 (0.71-0.82) |
|  | Favorable | 0.68 (0.63-0.73) |
| CMD-free | Unfavorable | 1 (Ref.) |
|  | Intermediate | 0.75 (0.72-0.78) |
|  | Favorable | 0.68 (0.65-0.71) |

The analyses were adjusted for age, sex, ethnicity, Townsend deprivation index, qualifications, employment, cholesterol, C-reactive protein, triglycerides, hypertension, use of aspirin, and use of insulin.

Table S11: Risk of all-cause mortality according to lifestyle factors category within CMDs status after adjusting PM 10, PM 2.5, and NO_2_.

| CMDs status | Lifestyle | HR (95% CI) ^b^ |
| --- | --- | --- |
| CMD | Unfavorable | 1 (Ref.) |
|  | Intermediate | 0.82 (0.75-0.90) |
|  | Favorable | 0.62(0.57-0.68) |
| CMD-free | Unfavorable | 1 (Ref.) |
|  | Intermediate | 0.78 (0.74-0.83) |
|  | Favorable | 0.56 (0.53-0.60) |

The analyses were adjusted for age, sex, ethnicity, Townsend deprivation index, qualifications, employment, cholesterol, C-reactive protein, triglycerides, hypertension, use of aspirin, and use of insulin.

Table S12: Risk of all-cause mortality according to lifestyle factors category within CMDs status after adjusting family history of CMDs.

| CMDs status | Lifestyle | HR (95% CI) ^b^ |
| --- | --- | --- |
| CMD | Unfavorable | 1 (Ref.) |
|  | Intermediate | 0.81 (0.75-0.88) |
|  | Favorable | 0.62 (0.57-0.67) |
| CMD-free | Unfavorable | 1 (Ref.) |
|  | Intermediate | 0.77 (0.73-0.82) |
|  | Favorable | 0.56 (0.53-0.59) |

The analyses were adjusted for age, sex, ethnicity, Townsend deprivation index, qualifications, employment, cholesterol, C-reactive protein, triglycerides, hypertension, use of aspirin, and use of insulin.

502,528 individuals recruited at baseline

41,491 **Excluded**

| Due to history of cancer at baseline; |
| --- |

461,037 individuals potentially available for study

104,070 **Excluded**

| Missing information on healthy lifestyles:  smoking 2,950; diet 1,550; physical activity 108,150; BMI 1,578 |
| --- |

356,967 individuals included finally

Figure S1: Participant flow diagram.
